# Fractionated mitochondrial magnetic separation for isolation of synaptic mitochondria from brain tissue

**DOI:** 10.1038/s41598-019-45568-3

**Published:** 2019-07-04

**Authors:** W. Brad Hubbard, Christopher L. Harwood, Paresh Prajapati, Joe E. Springer, Kathryn E. Saatman, Patrick G. Sullivan

**Affiliations:** 1 Spinal Cord & Brain Injury Research Center, University of Kentucky, Lexington, USA; 2 Department of Neuroscience, University of Kentucky, Lexington, USA; 3 Department of Physiology, University of Kentucky, Lexington, USA; 4 Lexington VAMC, Lexington, USA

**Keywords:** Protein purification, Diseases of the nervous system, Mitochondria

## Abstract

While mitochondria maintain essential cellular functions, such as energy production, calcium homeostasis, and regulating programmed cellular death, they also play a major role in pathophysiology of many neurological disorders. Furthermore, several neurodegenerative diseases are closely linked with synaptic damage and synaptic mitochondrial dysfunction. Unfortunately, the ability to assess mitochondrial dysfunction and the efficacy of mitochondrial-targeted therapies in experimental models of neurodegenerative disease and CNS injury is limited by current mitochondrial isolation techniques. Density gradient ultracentrifugation (UC) is currently the only technique that can separate synaptic and non-synaptic mitochondrial sub-populations, though small brain regions cannot be assayed due to low mitochondrial yield. To address this limitation, we used fractionated mitochondrial magnetic separation (FMMS), employing magnetic anti-Tom22 antibodies, to develop a novel strategy for isolation of functional synaptic and non-synaptic mitochondria from mouse cortex and hippocampus without the usage of UC. We compared the yield and functionality of mitochondria derived using FMMS to those derived by UC. FMMS produced 3x more synaptic mitochondrial protein yield compared to UC from the same amount of tissue, a mouse hippocampus. FMMS also has increased sensitivity, compared to UC separation, to measure decreased mitochondrial respiration, demonstrated in a paradigm of mild closed head injury. Taken together, FMMS enables improved brain-derived mitochondrial yield for mitochondrial assessments and better detection of mitochondrial impairment in CNS injury and neurodegenerative disease.

## Introduction

Mitochondria are small (0.5 to 2 μm) organelles that provide a majority of the cells’ energy in the form of adenosine triphosphate (ATP). In addition to providing the cell with ATP, mitochondria are responsible for regulating calcium homeostasis, cell signaling via metabolic shifts and apoptotic pathways. Furthermore, mitochondrial function is particularly important in the brain as the central nervous system (CNS) consumes around 20 percent of the body’s oxygen^1^. Specifically, mitochondria located in the neural synapse may undergo the highest bioenergetic demand in the brain as they supply energy needed during neurotransmission^2^. Synaptic mitochondria are highly involved in the regulation of neurotransmitter release^3^ and synaptic vesicle formation^4^. Other mitochondria in the brain (non-synaptic) can be derived from multiple cell types or locations, such as astrocytes, microglia, endothelial cells, and even neuronal soma. These perform essential roles, such as modification of microRNA activity^5^, and produce energy for various cellular activities.

The role of mitochondria in human disease continues to expand as new studies highlight their impact in a variety of medical disciplines^6^. Mitochondrial dysfunction is well established as a key pathological mechanism in neurodegenerative disease^7,8^. Moreover, synaptic mitochondria have been shown to be more vulnerable to calcium overload, aging and neurotrauma^9–12^, likely due to higher basal energy demand and pathological damage to the synapse. In neurodegenerative disorders^8,13,14^, significant changes in mitochondrial fission and fusion occur, contributing to variation in mitochondrial density at the synapse. Due to this specific susceptibility in neurodegeneration, it is crucial to obtain specific mitochondrial sub-populations, synaptic and non-synaptic fractions, to pinpoint functional changes. For functional measurements, synaptic mitochondria are extracted from synaptoneurosomes, which are formed during brain tissue homogenization.

**Figure 1:**
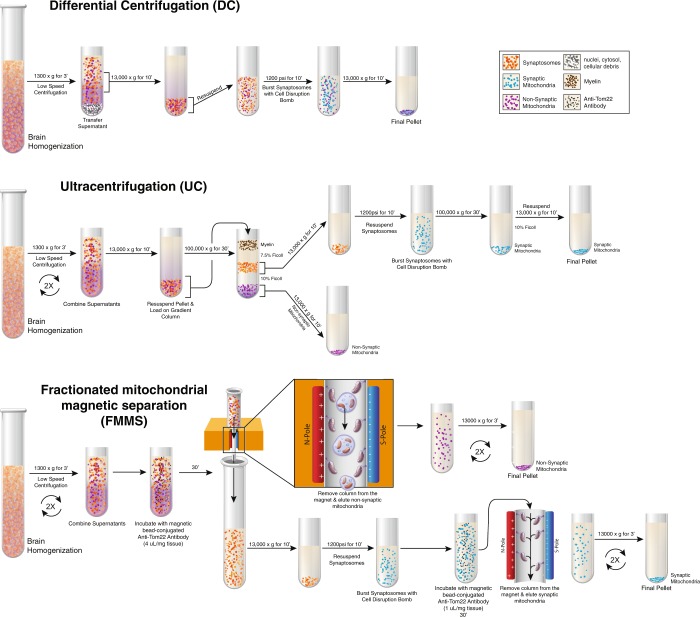
Schematic of mitochondrial isolation techniques. (Top) Workflow of differential centrifugation (DC) to isolate total mitochondria from mouse brain samples. (Middle) Workflow of density gradient ultracentrifugation (UC) to isolate synaptic and non-synaptic mitochondrial fractions from mouse brain samples. (Bottom) Workflow of fractionated mitochondrial magnetic separation (FMMS) technique to isolate synaptic and non-synaptic mitochondrial fractions from mouse brain samples. *Illustration by Matt Hazzard*, *University of Kentucky*, *Information Technology*.

Isolation techniques have been developed to keep mitochondria intact and functional, preserving their physical and biochemical characteristics. Two centrifugation methods for isolating mitochondria from CNS tissue are differential centrifugation (DC) and density gradient high speed/ultracentrifugation (UC). While these methods each have specific advantages that render them useful in different scenarios (Table 1), they have limitations for assessment of highly susceptible synaptic mitochondria, detailed below. The quickest and most inexpensive method for isolating mitochondria is DC, which utilizes centrifugation, ranging from 1000 × g to 15,000 × g, to isolate mitochondria from other cellular organelles (Fig. 1). DC yields total mitochondria, but also captures cellular debris that may interfere with downstream assays assessing mitochondrial activity^15^. This inability to isolate high-purity mitochondrial pellets or specific mitochondrial sub-populations creates the need for more intricate, albeit more expensive and time-consuming, techniques of mitochondrial isolation.

**Table 1:** Advantages and disadvantages of each technique described in this manuscript: differential centrifugation (DC), density gradient ultracentrifugation (UC), and fractionated mitochondrial magnetic separation (FMMS).

	DC	UC	FMMS
Yield	++	−	++
Purity	−	+	++
Function	+	+	+
Sub-Population Separation	−	+	+
Expense	++	−	+
Sensitivity	+	−	+

(−) technique does not have the capability or is a disadvantage. (+) technique has the capability, can perform well, or is an advantage. (++) technique has superior capability or has greater advantage compared to other methods. Sensitivity refers to detection of mitochondrial impairment.

Purified mitochondrial fractions have been obtained previously using Percoll gradient high speed centrifugation^16–18^. This technique requires a Percoll gradient step (at least 18,000 g spin^16^) to purify the non-synaptic, or “free,” mitochondrial fraction^18^. Additionally, this technique has been utilized to isolate non-synaptic mitochondria from small regions of the mouse brain, demonstrating relative high yield and purity^16^. While these reports using Percoll gradients do not include disruption of synaptoneurosomes and further isolation of synaptic mitochondria, our laboratory has used this technique to isolate both synaptic and non-synaptic mitochondrial populations^9,19^, though requiring higher speed centrifugation (30,400 g spin). In addition, these Percoll preparations result in phase bands of mitochondria rather than distinct mitochondrial pellets.

UC is a more specific isolation technique that utilizes exceedingly high speed centrifugation and buoyancy to allow for separation of non-synaptic and synaptic mitochondria^9,20,21^. In order to separate non-synaptic mitochondria from synaptoneurosomes, a Ficoll sucrose density gradient is used and free mitochondria are pelleted, as opposed to phase bands obtained during Percoll preparations (Fig. 1). While the UC approach allows for pure non-synaptic and synaptic mitochondria to be isolated, this method is expensive, requires technical proficiency and can be time-consuming when running many samples. A common issue with UC is being unable to obtain sufficient synaptic mitochondria yield for technical analysis from low initial tissue amounts, limiting its application to small brain regions such as the hippocampus in mice. In general, UC, similar to DC, requires many hands-on steps that can result in damage or loss of mitochondria (i.e. pipetting and supernatant transfer)^22^. Thus, there is a need for a mitochondrial isolation technique, such as affinity purification, that can preserve mitochondria yields and mitochondrial heterogeneity when using lower amounts of brain tissue.

The use of magnetically labeled antibodies for mitochondrial extraction has been shown to yield intact functional mitochondria from cell culture and various tissues^15,23–25^, but a protocol for isolating brain synaptic and non-synaptic mitochondria using this method has yet to be refined. Although the magnetic-activated cell sorting (MACS) system has been previously been utilized to purify synaptosomal mitochondria^26^, this procedure requires usage of density gradient UC. Magnetic mitochondrial isolation can be faster than UC techniques, depending on the specific application^27,28^.

In this study, we detail how to apply magnetic immunolabeling, using the MACS system, to target the Tom22 protein on the outer membrane of the mitochondria for synaptic and non-synaptic mitochondrial isolation. Fractionated mitochondrial magnetic separation (FMMS) optimization using magnetic labeling is described, including antibody titration to determine the optimal concentration for mitochondrial saturation. In addition, FMMS is compared to other mitochondrial isolation techniques to demonstrate its advantages (see Table 1). The current study is the first to demonstrate how the MACS system can be solely utilized to isolate, purify, and separate brain-derived non-synaptic and synaptic mitochondria without the use of UC (Fig. 1). This is widely relevant to any laboratory focused on assessing neurodegenerative disease or CNS injury without purchase of an ultracentrifuge. Finally, this protocol is applied to examine mitochondrial respiration in a paradigm of mild traumatic brain injury (TBI). Similar injury models demonstrate that mitochondrial fusion occurs after this injury, which increases mitochondrial heterogeneity^14^. We show that the newly developed FMMS technique can provide better resolution of mitochondrial function and higher mitochondrial yields from mouse brain tissue, eliminating the need to pool tissues from multiple animals.

## Results

### Protocol optimization - non-synaptic fraction

One modification to the manufacturers’ protocol was to adjust the concentration of tissue homogenate to buffer for antibody incubation and magnetic column loading. In the manufacturers’ protocol, 10 mL of buffer was recommended for 50 to 100 mg of brain tissue. To provide an exact ratio of buffer to brain tissue homogenized and to optimize using our own mitochondrial isolation buffer, we titrated in this recommended range, as to not overload the magnetic column, and tested yield and mitochondrial respiration (data not shown). We found that a concentration of 10 mL buffer per 75 mg of tissue was optimal for both outcome measures, which is within their recommendation.

**Figure 2:**
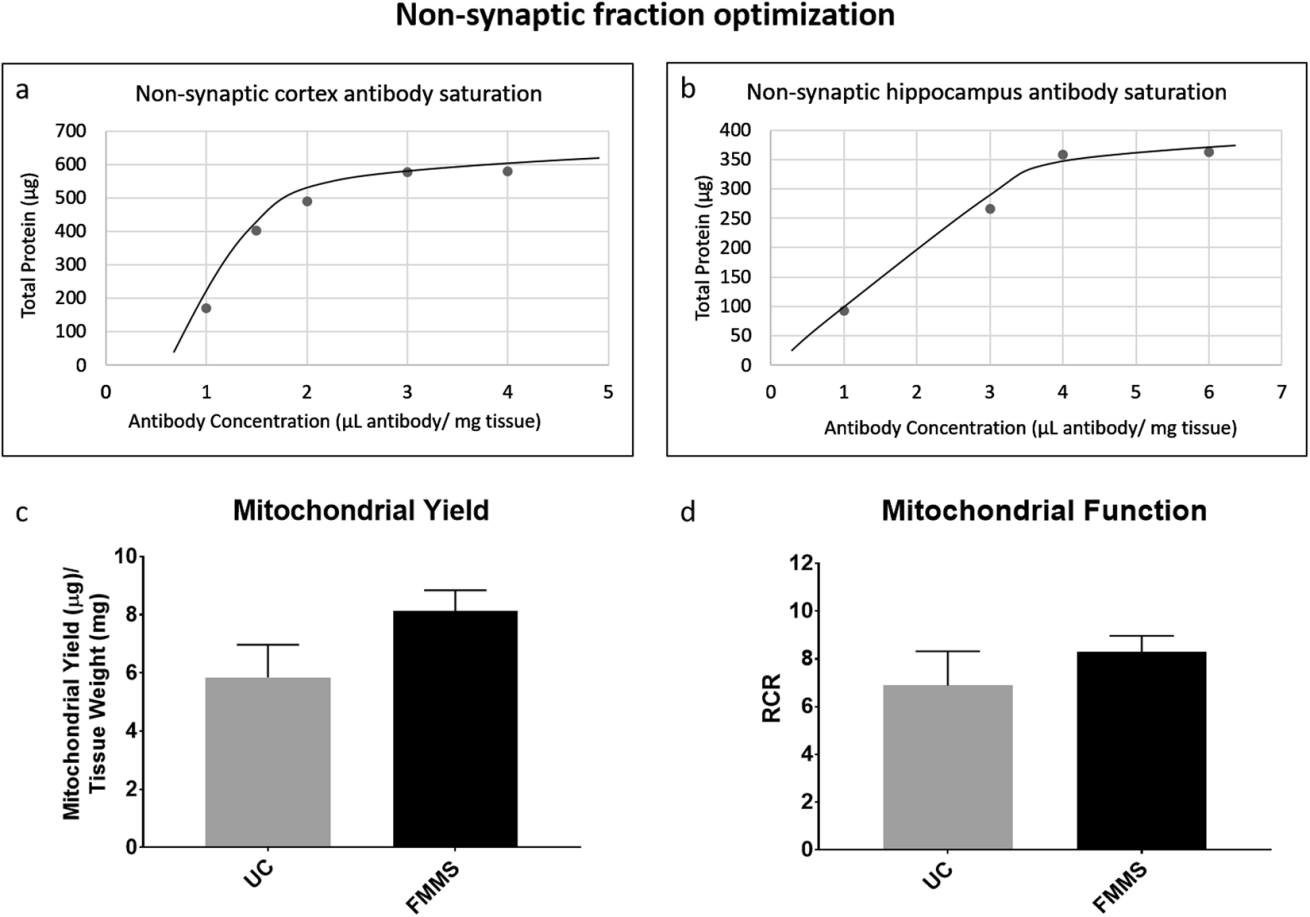
Non-synaptic fraction optimization (**a**) Cortical brain homogenates were centrifuged at low speed (1300 × g) and the supernatant was collected (procedure performed twice). This combined supernatant containing cortical non-synaptic mitochondria was incubated with 1, 1.5, 2, 3, and 4 µL antibody/mg tissue and total protein yield was calculated. (**b**) Hippocampal brain homogenates were centrifuged at low speed (1300 × g) and the supernatant was collected (procedure performed twice). This combined supernatant containing hippocampal non-synaptic mitochondria was incubated with 1, 3, 4 and 6 µL antibody/mg tissue and total protein yield was calculated. (**c**) Non-synaptic mitochondrial fractions were obtained from mouse hippocampus using the UC protocol and FMMS protocol. Mitochondrial yield (µg) was normalized to initial brain tissue amount (mg). (**d**) Non-synaptic mitochondrial fractions were obtained using the UC protocol and FMMS protocol. The Seahorse XFe24 Flux Analyzer was utilized to measure oxygen consumption rates (OCR) from these samples. Respiratory control ratio (RCR) was calculated by dividing State III OCR respiration values by State IV OCR respiration values. No significant difference was observed. N = 3–6/group. Bars + error bars correspond to Mean ± SEM.

A previous study by Franko, *et al*. reported that labeling mouse brain tissue with 0.5 μL antibody per mg of tissue resulted in a lack of saturation of mitochondria by magnetic beads^15^. To build upon these data and ensure that all free mitochondria were labeled, we ran assays of differing antibody to tissue concentrations measuring protein output. The range of antibody concentrations (1 to 6 μL of antibody per 1 mg of tissue) showed major differences in protein yield suggesting that the manufacturer recommended antibody concentration (0.5–1 μL/mg tissue) is not sufficient for mouse brain mitochondria saturation. In the cortex, we found that the antibody titration for non-synaptic mitochondria demonstrated a plateau effect, indicating saturation (Fig. 2a), while a similar trend was shown for non-synaptic hippocampus-derived mitochondrial protein (Fig. 2b). Based on these observations, it is recommended to use >3 μL/mg tissue for cortical non-synaptic mitochondria and >4 μL/mg tissue for hippocampal non-synaptic mitochondria, using the prescribed procedure in this manuscript.

For the FMMS technique, it is imperative to saturate the non-synaptic mitochondria with antibody to prevent free, unbound non-synaptic mitochondria from passing into the eluate during the first pass through the magnet (Fig. 1). This would lead to non-synaptic mitochondria in the synaptosomal fraction, biasing the mitochondrial population and leading to potentially inaccurate results. Thus, we increased the non-synaptic antibody concentration in our final protocol to 4 μL of magnetic antibody solution per mg of initial brain tissue to ensure total saturation. Increasing the antibody concentration for the non-synaptic pulldown also ultimately lead to a ~1.5x increase in mitochondrial yield compared to UC methods (Fig. 2c). Additionally, RCRs were comparable, even slightly higher, in mitochondria obtained from FMMS compared to those obtained by UC (Fig. 2d).

### Protocol optimization - synaptic fraction

**Figure 3:**
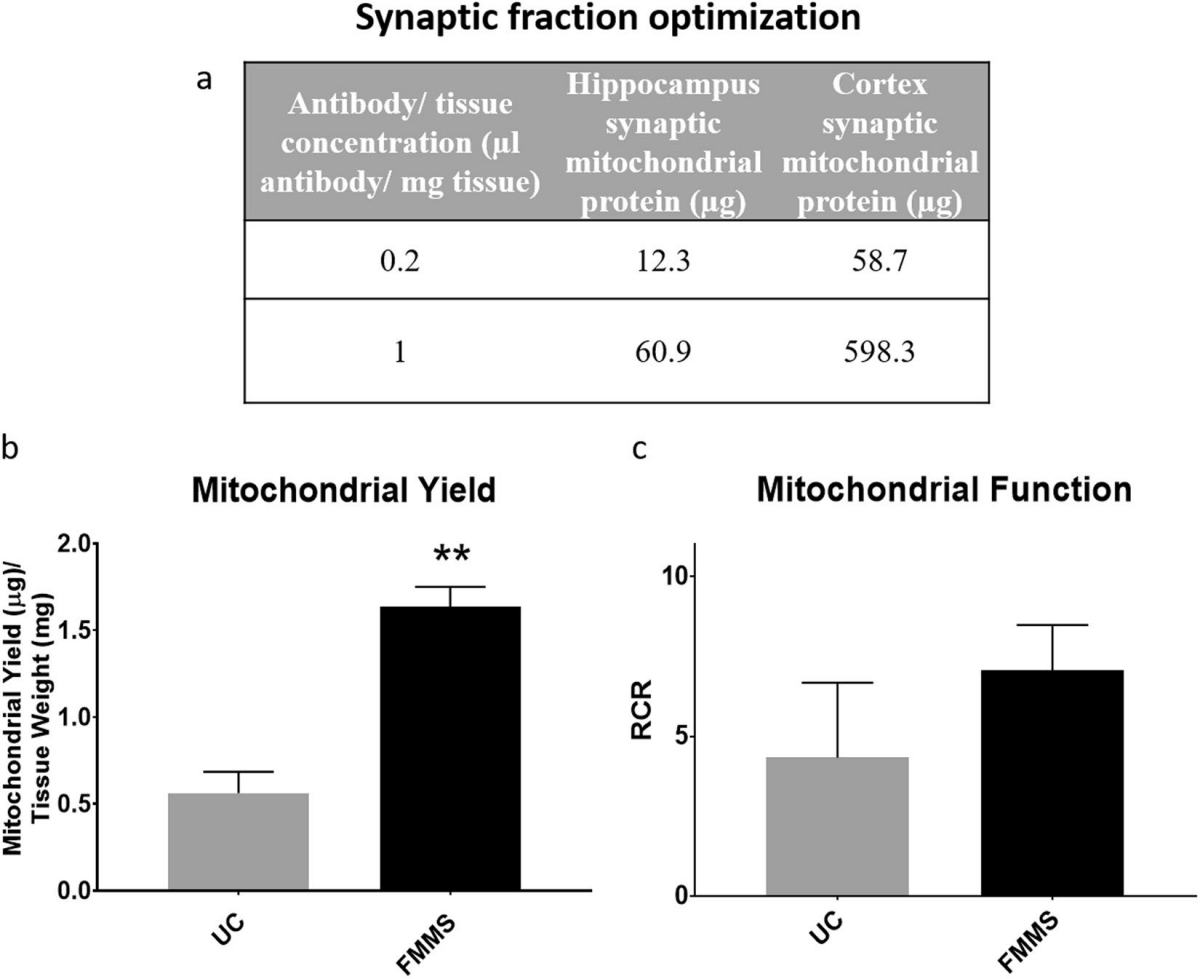
Synaptic fraction optimization. (**a**) Hippocampal and cortical synaptic mitochondrial fraction were separately saturated with 4 µL antibody/mg tissue and pulled through the magnetic column. The resulting eluate containing synaptoneurosomes was nitrogen bombed to release synaptic mitochondria. The supernatant containing hippocampal or cortical synaptic mitochondria was incubated with 0.2 or 1 µL antibody/mg tissue and total protein yield was calculated. (**b**) Synaptic mitochondrial fractions were obtained from mouse hippocampus using the UC protocol and FMMS protocol. Mitochondrial yield (µg) was normalized to initial brain tissue amount (mg). FMMS technique produced significantly higher (p < 0.01) levels of mitochondrial protein compared to UC methods. (**c**) Synaptic mitochondrial fractions were obtained using the UC protocol and FMMS protocol. The Seahorse XFe24 Flux Analyzer was utilized to measure oxygen consumption rates (OCR) from these samples. Respiratory control ratio (RCR) was calculated by dividing State III OCR respiration values by State IV OCR respiration values. No significant difference was observed. N = 3–6/group. Bars + error bars correspond to Mean ± SEM.

After determining the appropriate antibody concentration for non-synaptic mitochondria, we optimized antibody concentration for the synaptic fraction. After collecting the wash-through from the first magnet pass, synaptoneurosomes were pelleted by centrifugation. Afterwards, nitrogen disruption of synaptoneurosomes was completed to release synaptic mitochondria^29^. Due to the relatively smaller amount of synaptic mitochondria, a lower antibody concentration range was tested (0.2 to 1 μL of antibody per mg of tissue). As with the non-synaptic fraction, the yield was dependent on antibody concentration. A change in synaptic antibody concentration from 0.2 to 1 μL per mg of tissue resulted in a 5x increase in yield in the hippocampus and an even greater increase in yield in the cortex (Fig. 3a). Furthermore, FMMS yielded 3x more synaptic mitochondria compared to UC (Fig. 3b). Corroborating previous reports, RCR values of synaptic mitochondrial preparations were similar between FMMS and UC, although FMMS-derived samples demonstrated higher values (Fig. 3c)^28^. Thus, the FMMS protocol isolates intact and functional synaptic mitochondria from the mouse brain.

### Mitochondrial purity after FMMS and UC methods

**Figure 4:**
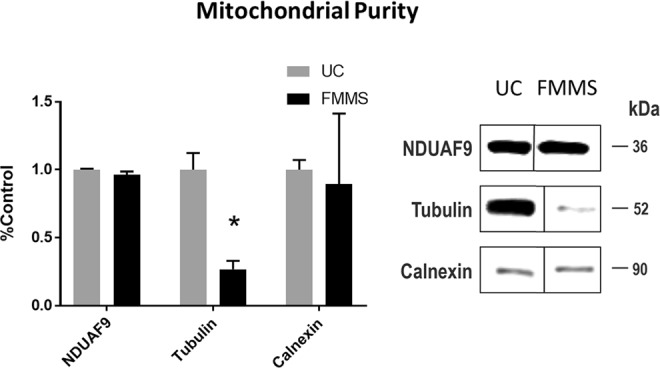
Markers of mitochondrial fraction purity after either UC or FMMS procedures. (Left) Protein levels, obtained by western blot, were normalized to UC values. While NDUAF9 and calnexin levels were not significantly different between the groups, tubulin levels were significantly lower after FMMS methods compared to UC methods. (Right) Representative western blot for all markers. These blots were cropped from different parts of the same gel for clarity. Full-lengths blots are provided in the Supplementary material (Fig. S1). N = 5–7/group. *p < 0.05 compared to UC. Bars + error bars correspond to Mean ± SEM.

Previous studies have shown that DC-derived mitochondrial samples contain cellular debris that can be eliminated with an additional magnetic separation step^15,30^. We evaluated our UC- and FMMS-derived synaptic fractions using three different markers, NDUAF9 (mitochondria), tubulin (cytosol), and calnexin (mitochondria-associated membrane). We found that, as expected, both NDUAF9 and calnexin levels were similar for the two isolation methods. However, tubulin levels were significantly lower (p < 0.05) in the FMMS-derived sample, indicating a higher purity can be obtained compared to UC (Fig. 4).

### Methodological sensitivity for detection of mitochondrial dysfunction after CNS injury

**Figure 5:**
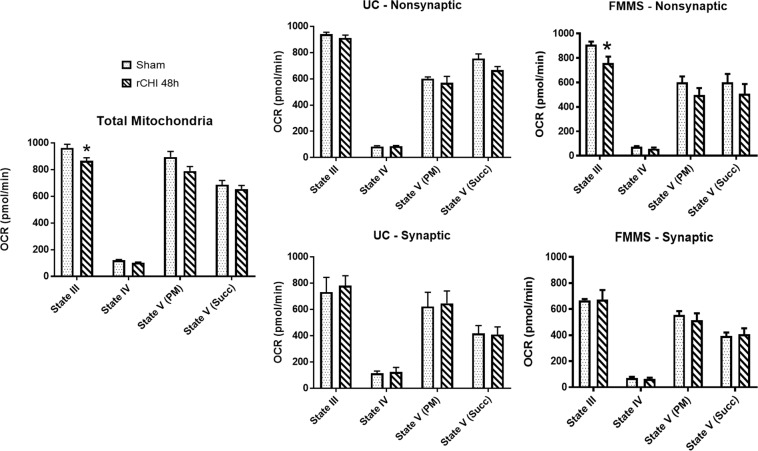
Application of FMMS in a model of repeated closed head injury. Mice were given either a repeated CHI (rCHI) at a 48 h interval or sham procedure. At 48 h after the final CHI, bilateral hippocampus was extracted and homogenized for mitochondrial respiration assessment. (Left) Total mitochondria obtained through DC methods demonstrated a State III OCR decrease in the repeated CHI group compared to Sham. This data was modified from previously published work^31^. (Middle) Non-synaptic and synaptic fractions were obtained by UC procedures. Neither fraction showed any significant differences between repeated CHI and Sham groups. (Right) Non-synaptic and synaptic fractions were obtained by FMMS. While the synaptic fraction did not show any significant differences between repeated CHI and Sham groups, State III OCR was lower in the non-synaptic fraction of the repeated CHI group compared to Sham. N = 6/group. *p < 0.05 compared to Sham. Bars + error bars correspond to Mean ± SEM.

In applying this protocol, we used a model of repeated mild TBI that has previously shown mitochondrial deficits^31^. Building upon data demonstrating a State III respiration decrease after injury (t = 2.843; p = 0.0294) in total (DC) mitochondria^31^, we employed UC and FMMS protocols to assess synaptic and non-synaptic fractions. We expect that UC or FMMS can resolve which mitochondrial fraction displays the highest level of dysfunction. A disadvantage of the UC method is that it required both hippocampi from three mice be *pooled* in order to achieve enough synaptic mitochondria protein to run the respiration assay in triplicate. In bilateral hippocampus (40 mg wet tissue), we did not observe any significant changes in State III respiration from either synaptic or non-synaptic mitochondrial fractions using the UC isolation procedure. In contrast, FMMS could be performed on hippocampal tissue (35 mg wet weight) from individual mice exposed to the same injury paradigm. Analysis of the FMMS-derived non-synaptic mitochondrial fraction revealed a statistically significant decrease (t = 2.521; p = 0.0284) in State III function compared to the sham group (Fig. 5). This suggests that FMMS, in addition to increasing yield, produces a higher level of resolution, potentially by capturing a higher percentage of damaged mitochondria that would be lost during the UC procedure.

## Discussion

The current standard method for isolating synaptic and non-synaptic mitochondrial fractions is UC with the use of a sucrose density gradient. While UC effectively separates these fractions in large brain tissue samples, there are limitations in obtaining sufficient synaptic mitochondrial yield from small amounts (<60 mg) of brain tissues. In addition to the lack of mitochondrial yield, there is concern that UC preparations result in the loss of dysfunctional mitochondria thereby making it less sensitive for detecting changes associated with brain pathology. In contrast, FMMS can separate mitochondrial fractions in a relatively quick and effective manner, resulting in a higher yield of both functional and, in the context of neurological disease or injury, dysfunctional mitochondria with increased purity. Importantly, FMMS allows isolation of synaptic and non-synaptic mitochondria from small brain regions, such as mouse hippocampi, and provides increased sensitivity for detection of mitochondrial dysfunction relative to UC.

In this study, we optimized the use of the MACS system with magnetic anti-Tom22 antibodies for isolation of synaptic and non-synaptic mitochondria from the mouse hippocampus and cortex. Secondly, we modified the manufacturer’s protocol for extracting tissue amounts lower than the recommended range of 50–100 mg tissue. The optimal antibody to tissue concentration was determined by measuring non-synaptic mitochondrial protein yield until the saturation point was reached (Fig. 2). Additionally, it was crucial to reach non-synaptic fraction saturation to prevent non-synaptic mitochondrial crossover into the synaptic fraction. While the non-synaptic cortical fraction had a saturation point around 3 µL antibody per mg of tissue, the hippocampal non-synaptic fraction saturated at 4 µL antibody per mg of tissue (Fig. 2b). Hippocampal tissue could require a higher antibody concentration to saturate non-synaptic mitochondria either due to a higher cell density or higher mitochondria number per cell as compared to the cortex. Given these observations, specific brain regions should be independently optimized for antibody concentration. Antibody concentrations used for the synaptic mitochondrial fraction (Fig. 3a) can be adjusted to achieve sufficient mitochondria needed for downstream assays.

One limitation of UC methods is the inability to sustain initial mitochondrial protein levels due to loss of mitochondria during multiple pipetting steps^32^ and density gradient fractionation. In pathological settings such as neurodegenerative disease, traumatic injury, or ischemic insult, mitochondria can be damaged or undergo fusion or fission^13,14,33^ resulting in a change in density that could preclude their capture during UC. This issue is mitigated by FMMS as, at mitochondrial saturation, every mitochondrion that expresses Tom22, a component of the translocase of the outer mitochondrial membrane, is theoretically labeled. Of course, ruptured mitochondria without an outer membrane would not be captured using this technique. All samples in this report include mitochondria that are bound to these magnetic microbeads. Due to the small size (50 nm), biodegradability, and non-toxic nature of these microbeads (Miltenyi Biotec), they are compatible with all downstream applications and do not need to be removed which is an added advantage of FMMS. We corroborate this by showing comparable RCR values of FMMS-derived mitochondria compared to UC-derived mitochondria (Figs 2 and 3)^15^.

We show that mitochondrial protein levels greatly increase when employing FMMS compared to UC (Fig. 3b). Non-synaptic mitochondrial yield is similar to a previously published report on a Percoll gradient technique isolating “free” mitochondria from mouse striatum^16^, though this did not include subsequent synaptic mitochondria isolation. Previous reports detailing synaptic mitochondrial respiration within subregions of the mouse brain require pooling of tissues from multiple mice to create one sample^34–36^. A previous study has shown that the amount of mitochondrial protein was ~4x more using the MACS system compared to UC methods^28^, which is corroborated in the current study (Fig. 3b). Our current technique would eliminate the need for animal pooling due to the much higher (3x more) mitochondrial protein yields in small brain regions compared to UC methods. In addition to animal reduction, this also would reduce variability inherent with pooled tissues while increasing experimental/statistical power by allowing for animal-to-animal correlations with other outcomes, such as behavioral tests. FMMS showed a 45% increase in non-synaptic yield and a 200% increase in synaptic yield compared to UC in hippocampal tissue (Figs 2c and 3b). This increase in yield highlights the utility of our FMMS protocol for examining mitochondrial function in small brain regions.

Mitochondrial preparation using UC has the potential for selective loss of dysfunctional mitochondria, which are critical for evaluation of brain pathology. This decreases the mitochondrial heterogeneity, leading to an incomplete view of mitochondrial function (or dysfunction). As an example, mitochondrial dysfunction, initiated by repeated mild TBI^31^, was detected in a total mitochondria DC preparation as a decrease in State III respiration, but was not apparent after synaptic/non-synaptic fractionation by UC (Fig. 5). However, when using FMMS, significant State III respiration decrease was again noted and was identified to preferentially involve the non-synaptic fraction, suggesting that damaged mitochondria are preserved using this method. Therefore, FMMS provides increased sensitivity to pinpoint regional and temporal mitochondrial changes in neurodegenerative disorders or after CNS injury. It is important to note that maintaining mitochondrial heterogeneity with FMMS would provide greater resolution in detecting therapeutic efficacy of mitochondrial-directed treatments in various neurological diseases.

The quality and purity of MACS-derived mitochondria, with respect to amounts of endoplasmic reticulum (ER) and nucleus contamination, has been shown to be comparable to the UC method^28^. A subsequent study reported less nuclear contamination with the MACS system than with DC and UC methods^37^. Additionally, combining DC with MACS separation achieves higher purity (less cytosol, ER, and nuclear contaminates) compared to either DC or MACS mitochondrial isolation alone^30^. Consistent with this study, we show that FMMS results in significantly lower amounts of cytosolic tubulin compared to UC methods (Fig. 4). Overall, FMMS-derived mitochondrial samples have higher purity, with less cytosolic and potentially less ER contamination, compared to UC-derived mitochondrial samples. While MACS separation is reliable for respiration assays, it has limitations with other sensitive assays such as lipidomics due to some amounts of ER and nuclear fraction contamination^38^. An additional limitation is that the MACS kits and antibodies are expensive, especially compared to DC methods, although much less than the cost of an ultracentrifuge. Furthermore, antibody titration is required for each brain region utilized as it is crucial to reach non-synaptic fraction saturation to prevent non-synaptic mitochondrial crossover into the synaptic fraction.

The implications of this new and improved technique are far-reaching in the fields of CNS injury and neurodegenerative disease^39^. Using the current protocol, researchers will be able to harness the power of FMMS to assay small, but crucial, brain regions in individual mice providing a degree of regional specificity that was not previously possible. For example, FMMS could be utilized to assay hippocampal sub-regions important in aging^12^, Alzheimer’s Disease (AD)^11^ and neurotrauma^40^, or to assay mitochondria from the amygdala, a region associated with post-traumatic stress disorder (PTSD)^41^ and other stress-related neuropsychiatric disorders^42^. Basal ganglia in the mouse could potentially be examined now using FMMS, yielding new insights relevant to neurological disorders such as Parkinson’s Disease (PD)^43^ and Huntington’s Disease (HD)^44–46^. Furthermore, using FMMS could reveal greater mitochondrial dysfunction than determined previously, based on higher sensitivity of the technique.

## Conclusion

In this study, we identified the optimal procedures for isolating synaptic and non-synaptic mitochondria from the hippocampus and cortex using FMMS. The FMMS protocol presents several benefits over traditional UC isolation techniques. Advantages of this system (Table 1) include the ability to derive synaptic and non-synaptic mitochondrial fractions from low amounts of neuronal tissue (35 mg), while producing similar mitochondrial respiration profiles. Furthermore, FMMS yields significant increases in mitochondrial protein compared to UC techniques. Finally, using the magnetic system preserves the ability to measure mitochondrial respiration from a heterogeneous population of mitochondria, including damaged and undamaged mitochondria. This concept is highlighted when applying FMMS in a model of repeated mild TBI. This technique has wide-ranging implications in the field of brain metabolism and neurodegenerative disease. With FMMS, researchers can gain greater understanding of how mitochondria within specific brain subregions are altered after pathological insult.

## Materials and Methods

### Reagents - UC

Fresh mouse brain tissue sample

Sucrose (Sigma, cat. no. S8501)

Mannitol (Sigma, cat. no. M9546)

Bovine Serum Albumin (BSA; Sigma, cat. no. A7511)

HEPES (Sigma, cat. no. H0887)

Ethylene-bis (oxyethylenenitrilo) tetraacetic acid (EGTA; Sigma, cat. no. E0396)

Ficoll (Sigma, cat. no. F5415)

Trizma Base (Sigma, cat. no. T6066)

### Reagents - FMMS

Fresh mouse brain tissue sample

Sucrose (Sigma, cat. no. S8501)

Mannitol (Sigma, cat. no. M9546)

Bovine Serum Albumin (BSA; Sigma, cat. no. A7511)

HEPES (Sigma, cat. no. H0887)

Ethylene-bis (oxyethylenenitrilo) tetraacetic acid (EGTA; Sigma, cat. no. E0396)

### Equipment – UC

Teflon-glass dounce homogenizer

1.5 mL micro centrifuge tubes

2 mL micro centrifuge tubes

Eppendorf 5424 centrifuge

Ultracentrifuge Optima XE-90 (Beckman Coulter, Fullerton, CA)

SW 55 Ti Swinging-Bucket Rotor

Cell disruption vessel (Parr Instrument Company, cat. no. 4635).

Nitrogen tank

### Equipment – FMMS

Teflon-glass dounce homogenizer

1.5 mL micro centrifuge tube

2 mL micro centrifuge tube

15 mL conical vial

Eppendorf 5424 centrifuge

Mitochondria Isolation Kit for mouse tissue, which includes anti-Tom22 microbeads, MACS separation LS Columns, and magnetic Quadro MACS Separator (Miltenyi Biotec, cat. no. 130-097-040).

Cell disruption vessel (Parr Instrument Company, cat. no. 4635)

Nitrogen tank

The Belly Dancer®, Belly Dancer shaker (Denville Scientific)

### Reagent Preparation – UC

Isolation Buffer (IB): 215 mM mannitol, 75 mM sucrose, 0.1% BSA, 20 mM HEPES, 1 mM EGTA, Adjusted pH 7.2 with KOH

Ficoll stock (final concentration): 12% Ficoll, 0.3 M Sucrose, 10 mM Tris and 0.2 mM EGTA

10% Ficoll: 43 mL of Ficoll Stock bring to 50 mL volume with IB

7.5% Ficoll: 33 mL of Ficoll Stock bring to 50 mL volume with IB

### Reagent Preparation – FMMS

Isolation Buffer (IB): 215 mM mannitol, 75 mM sucrose, 0.1% BSA, 20 mM HEPES, 1 mM EGTA, Adjusted pH 7.2 with KOH

### UC Mitochondrial Isolation

- After CO_2_ euthanasia and decapitation, rapidly remove the brain and dissect selected brain region(s) and rinse thoroughly with IB (remove blood and other debris).
- Mechanically homogenize the tissue in a 10 mL Teflon-glass dounce homogenizer with 2 mL (~10X volume of IB to tissue) of IB (3–5 vertical strokes).
  Critical Step: Keep homogenate and resuspensions on ice or at 4 °C as higher temperatures may result in mitochondrial denaturation. Make sure to prevent air bubbles during homogenization.
- Centrifuge the homogenized sample for 3 minutes at 1300 rcf at 4 °C and transfer the supernatant to a 2 mL micro centrifuge tube (mitochondria remain in the supernatant).
- Resuspend the pellet in 1 mL of IB, repeat step (c), and combine supernatants for maximum yield (may result in two tubes per sample).
- Centrifuge tube (s) at 13,000 rcf for 10 minutes at 4 °C.
- Meanwhile, prepare the density gradient in ultracentrifuge tubes by adding 2 mL of 10% Ficoll solution followed by gently adding 2 mL of 7.5% Ficoll solution (make sure a distinct interface can be seen between the two solutions in the tube).
- Discard the supernatant and resuspend the crude mitochondrial pellet in 500 μL of IB.
- Add the mitochondrial sample over top of the prepared Ficoll double layered solution and centrifuge at 100,000 rcf for 30 minutes at 4 °C using ultracentrifuge to obtain synaptoneurosomes and non-synaptic mitochondria.
- Collect synaptoneurosomes by pipetting the layer from the interface of the two Ficoll densities and non-synaptic mitochondria from the pellet.
  Critical Step: When collecting synaptosomal layer, take as little Ficoll solution as possible in the pipette. Transferring a large amount of Ficoll solution will result in an inability of synaptoneurosomes to pellet.
- Transfer the synaptosomal fraction to a 2 mL centrifuge tube with 4X diluted IB and centrifuge at 13,000 rcf for 10 minutes at 4 °C.
- Discard the supernatant and resuspend the pellet in 400 μL of IB.
- Place the synaptosomal fraction in a nitrogen cell disruptor at 1200 PSI for 10 minutes at 4 °C to rupture the synaptoneurosomes.
- Take the resulting solution into a 1.5 mL centrifuge tube and centrifuge at 13,000 rcf for 10 minutes at 4 °C.
- Meanwhile, prepare 3.5 mL of 10% Ficoll solution in ultracentrifuge tubes.
- Discard the supernatant and resuspend the crude mitochondrial pellet in 500 μL of IB.
- Add the mitochondrial sample over top of the prepared 10% Ficoll solution and centrifuge at 100,000 rcf for 30 minutes at 4 °C using ultracentrifuge to purify synaptic mitochondria from other debris (the synaptic mitochondria will reside in the pellet).
- Separately resuspend both synaptic and non-synaptic fraction pellets in 600 μL of IB (1.5 mL micro centrifuge tubes) and centrifuge at 13,000 rcf for 10 minutes at 4 °C.
- Discard the supernatant and resuspend the pellets in an appropriate volume of IB (optimal volume results in around 10 μg of mitochondria per μL of solution).
- Both samples (non-synaptic and synaptic fractions) are now ready for protein analysis.

### FMMS Mitochondrial Isolation

#### Non-synaptic mitochondrial isolation

Our protocol was adapted from the manufacturer’s mitochondrial isolation protocol, incorporating aspects of previously published work^47^. This isolation uses centrifugation steps followed by magnetic separation, similar to previous methodology^30^. Mouse brain tissues were rapidly dissected from naïve C57BL/6J male mice (Jackson Laboratories, Bar Harbor, Maine). Samples were kept at 4 °C on ice and all reagents were previously stored at 4 °C to prevent degradation of the mitochondria. Mitochondria were isolated from brain tissue samples using mouse mitochondrial isolation kits (Miltenyi Biotec). A schematic of the procedure is shown in Fig. 1.

- After CO_2_ euthanasia and decapitation, rapidly remove the brain and dissect selected brain region(s) and rinse thoroughly with IB (remove blood and other debris).
- Weigh the tissue and record mass for later use.
- Mechanically homogenize the tissue in a 10 mL Teflon-glass dounce homogenizer with 2 mL of IB (3–5 vertical strokes).
  Critical Step: Keep homogenate and resuspensions on ice or at 4 °C as higher temperatures may result in mitochondrial denaturation. Make sure to prevent air bubbles during homogenization.
- Centrifuge the sample for 3 minutes at 1300 rcf at 4 °C in a 2 mL micro centrifuge tube and transfer the supernatant to a 15 mL conical vial (mitochondria remain in the supernatant).
- Resuspend the pellet in 1 mL of IB, repeat step (d), and combine supernatants for maximum yield.
- Add IB at a concentration of 1 mL for every 7.5 mg of tissue weight to bring to volume and prevent subsequent magnetic column overloading.
- Add anti-Tom22 magnetic antibody at a concentration of 4 μL for every 1 mg of tissue weight (based on results in this manuscript).
  Critical Step: Specific brain regions may require more or less antibody for optimal isolation. We recommend performing an antibody titration to determine this.
- Incubate the sample at 4 °C for 30 minutes while ensuring constant rotation (i.e. Belly Dancer) for proper mixture.
- Set up the complete MACS column and wash with 3 mL of IB (Complete column: LS column, 30 μm filter, and MACS separator).
- Add the sample to the complete MACS column in 3 mL aliquots. Allow each aliquot to completely pass the column before subsequent addition.
  Critical Step: Load the column slowly to avoid air bubbles. Air bubbles may lead to decreased mitochondrial yield.
- Collect the eluate, which contains synaptoneurosomes, for downstream processing.
- Wash the column three times with 3 mL of IB to remove contaminants.
- Discard the 30 μm filter and remove the column from the separator to place above a 15 mL conical vial (for ease of transfer).
- Add 1.5 mL of IB to the column and plunge the sample quickly and aggressively.
- Transfer the isolated sample to a 1.5 micro centrifuge tube and centrifuge at 13,000 rcf for 10 minutes at 4 °C.
- Remove the supernatant and resuspend the sample in 1 mL of IB.
- Repeat step o and resuspend in an appropriate volume of IB for protein analysis.
- Non-synaptic sample is ready for protein quantification.

### Synaptic mitochondrial isolation

- Centrifuge the eluate (step k (from FMMS Non-synaptic Mitochondrial Isolation)) from the non-synaptic pulldown at 13,000 rcf for 10 minutes at 4 °C.
- Discard the supernatant and resuspend the pellet in 500 µL of IB.
- Place the samples in a nitrogen cell bomb at 1200 PSI for 10 minutes to rupture the synaptoneurosomes.
- Add anti-Tom22 magnetic antibody at a concentration of 1 μL for every 1 mg of original tissue (this may need to be optimized depending on the specific brain region).
- Level the samples off at 2 mL and incubate the sample at 4 °C for 30 minutes while ensuring constant rotation (i.e. Belly Dancer) for proper mixture.
- Complete the pulldown identically to steps i, j, l, m, n (from FMMS Non-synaptic Mitochondrial Isolation).
- Complete post pulldown processing identically steps o, p, q (from FMMS Non-synaptic Mitochondrial Isolation).
- Synaptic fraction is ready for protein quantification.

Protein was quantified using a BCA analysis kit (Pierce, Cat # 23,227) and the absorbance was measured at 560 nm on a Biotek Synergy HT plate reader (Winooski, Vermont). Anti-TOM22 magnetic microbeads present in our samples did not contribute to over-estimation of protein.

### Measurement of oxygen consumption rates with Seahorse XFe24 flux analyzer

The mitochondrial bioenergetic measurements were carried out using a Seahorse XFe24 Extracellular Flux Analyzer (Agilent Technologies, USA), which determines the bioenergetics of mitochondria by measuring the Oxygen Consumption Rates (OCR) during various states of respiration. The OCR were measured in the presence of different substrates, inhibitors and un-couplers of the Electron Transport Chain (ETC) using previous methods with slight modifications^31^. The stocks used for the assays were 500 mM pyruvate, 250 mM malate, 30 mM adenosine diphosphate (ADP), and 1 M succinate (pH for all were adjusted to 7.2). Stocks of 1.26 mM oligomycin A, 1 mM carbonyl cyanide 4-(trifluoromethoxy) phenylhydrazone (FCCP), 1 mM rotenone were prepared in ethanol and used for assays. As per the instructions from the XFe24 Extracellular Flux kit, the sensor cartridge was hydrated overnight at 37 °C. The injection ports A to D of the sensor cartridge were then loaded with 75 μL of different combinations of the above substrates/inhibitors/uncouplers as follows. Before loading, the stocks were diluted appropriately in the respiration buffer (RB) to get the final concentrations in the respiration chamber of 5 mM pyruvate/ 2.5 mM malate/ 1 mM ADP (via Port A), 1 μM oligomycin A (via Port B), 4 μM FCCP (via Port C) and 0.1 μM rotenone/10 mM of succinate (via Port D) starting with the initial volume of 525 μL RB in the chamber and accounting for the addition of volume with every injection through ports A to D. Once loaded, the sensor cartridge was placed into the Seahorse XFe24 Flux Analyzer for automated calibration.

Seahorse Standard XFe24 assay plates were used for loading mitochondria. The MACS-purified non-synaptic and synaptic mitochondria were diluted to 5 μg and 10 μg, respectively, per 50 μL in RB and 50 μL was loaded in each well. The UC-purified non-synaptic and synaptic mitochondria were diluted to 2.5 μg and 5 μg, respectively, per 50 μL in RB and 50 μL of this solution was loaded in each well. The assay plates were centrifuged at 3,000 rpm for 4 min at 4 °C to adhere the mitochondria to the bottom of the wells. After centrifugation, 475 μL of RB (pre-incubated to 37 °C) was added without disturbing the mitochondrial layer to obtain a final volume of 525 μL per well. After the instrument calibration with the sensor cartridge was complete, the utility plate was replaced by the plate loaded with mitochondria for bioenergetics analysis. Briefly, it involved cyclic steps of mixing, sequential injections of substrates and inhibitors via Ports A thru D, mixing, equilibration, and measurement of the OCR and pH through fluorimetric optical probes. The data output consisted of State III respiration in the presence of pyruvate, malate and ADP (Port A) followed by State IV rate in presence of oligomycin A (Port B). Subsequently, uncoupled respiration State V_PM_ (State V_1_) in the presence of FCCP (Port C) and State V_Succ_ (State V_2_) in the presence of rotenone/ succinate (Port D) were assayed, respectively. Respiratory control ratio (RCR) was calculated by dividing State III respiration values by State IV respiration values.

### Methodology for Repeated Mild TBI

#### Animals and experimental design

All animal procedures were approved by the Institutional Animal Care and Use Committee at the University of Kentucky and complied with the National Institutes of Health Guide for the Care and Use of Laboratory Animals. The Division of Laboratory Animal Resources at the University is accredited by the Association for the Assessment and Accreditation for Laboratory Animal Care, International (AAALAC, International) and all experiments were performed within its guidelines. All data were analyzed and reported according to ARRIVE guidelines. Young adult (7–9 week old; 22–26 g) C57BL/6 J male mice (Jackson Laboratories, Bar Harbor, Maine) were acclimated for a one-week period to the vivarium where they were housed (five per cage) in a 14 h/10 h light/dark cycle with food and water available ad libitum. Mice were then randomly assigned to two groups: repeated closed head injury (CHI) at a 48 h interval and sham. Animals (n = 6/group) were euthanized before mitochondrial isolation at 48 h after the final sham injury or CHI. For all assays, technical triplicates were included for each sample. Data analysis was performed blinded to treatment groups.

#### Closed head injury

Experimental CHI was induced following a previously described procedure^48^. Mice were anesthetized with 2.5% isofluorane delivered via a nose cone and the head of each mouse was fixed between two zygomatic cuffs stabilized in a stereotaxic frame. A pneumatically controlled cortical impact device (TBI-0310 Impactor, Precision Systems and Instrumentation, Fairfax Station, VA) with a 5 mm diameter, cushioned tip was programmed to deliver a 2.0 mm impact at 3.5 m/s with a 500 ms dwell time. The posterior edge of the tip was aligned at the Lambda suture (approximately Bregma level −5mm). The diameter of the tip (5 mm) is such that the anterior edge of the tip meets the Bregma suture (0 mm Bregma level). The second injury was induced at the same location. This impact was previously characterized such that a single injury would result in minimal gliosis or cell death without resulting in skull fracture^48^. The scalp was sutured using vicryl sutures containing antibiotics (Ethicon, Cincinnati, OH). Sham-injured animals underwent identical anesthesia and surgical procedures without receiving an impact on the final day of CHI. All mice were monitored on a heating pad until they became ambulatory. Additionally, mice were evaluated at 1–3 h and 24 h after each injury, followed by daily inspections. All mice were required to maintain 85% of their starting weight in order to receive repeated head injury. However, no mice needed to be removed from the study. After euthanasia at 48 h post-injury, mitochondria were extracted by either by DC (based on previous studies^31,47^), UC (as in 1.7), or FMMS (as in 1.8). For UC methods, brain regions were pooled from three mice for one data point to achieve a measurable synaptic mitochondria fraction. Additionally, respiration assays were completed according to Seahorse XFe24 methods detailed above.

### Western blots – sample purity

Mitochondrial fractions, obtained using UC or FMMS protocols, were lysed in RIPA buffer (Cat# R0268) and protein concentration determined using the Pierce BCA Protein Assay Kit (ThermoFisher). Equal amounts of proteins were then separated using 4–16% Mini-PROTEAN® TGXTM precast gels (Cat# 456–1106, Bio-Rad). After transferring onto nitrocellulose membranes, the blots were incubated with 5% nonfat milk for 1–2 hr before an overnight incubation with primary antibodies at 4 °C. The primary antibodies used included anti-calnexin (Cat # ab22595; Abcam), anti-beta tubulin (Cat # ab6046; Abcam) and NDUAF9 (Cat # 459100, Invitrogen). Blots were then incubated with Affinipure peroxidase-conjugated goat anti-mouse IgG and goat anti-rabbit IgG (Cat # 115–035–166 and 111-035-144, respectively; Jackson ImmunoResearch laboratories, Inc.) secondary antibodies. Immunoreactive signals were visualized on x-ray film using a SuperSignal™ West Pico PLUS Chemiluminescent Substrate (Cat # 32209, Pierce). The immunoreactive bands on the x-ray film were scanned using ODYSSEY CLX (LI-COR) and immunoreactive signals quantified using Image Studio Ver 5.2.

### Statistics

Statistical analysis was performed using Graph Pad Prism (GraphPad Software, CA, USA). For all analyses, the significance of differences among groups was set at p < 0.05. Triplicate data was averaged for each data point. For each measure, data were measured using interval/ratio scales. The Brown-Forsythe and Bartlett’s tests were performed to ensure homogeneity of variance. Furthermore, the Shapiro-Wilk test was completed to ensure normality. As these criteria were met for all experimental data, parametric statistics were employed for all data analyses. For all mitochondrial assessments, data sets were evaluated using an unpaired t-test to determine significance between the groups.

## Supplementary information

Supplementary western blot
