# Supplementary material for: Fractionated mitochondrial magnetic separation for isolation of synaptic mitochondria from brain tissue
Source: Sci Rep. 2019 Jul 4;9:9656. doi: 10.1038/s41598-019-45568-3 (PMC6609636; doi:10.1038/s41598-019-45568-3)
Supplement: Supplementary file 1 — Supplementary western blot [file 41598_2019_45568_MOESM1_ESM.docx]

Fractionated mitochondrial magnetic separation for isolation of synaptic mitochondria from brain tissue

#W. Brad Hubbard^1,2,3^, #Christopher L. Harwood^1,2^, Paresh Prajapati^1,2^, Joe E. Springer^1,2^ Kathryn E. Saatman^1,3^, *Patrick G. Sullivan^1,2,4^

^1^Spinal Cord & Brain Injury Research Center, University of Kentucky

^2^ Department of Neuroscience, University of Kentucky

^3^ Department of Physiology, University of Kentucky

^4^ Lexington VAMC

#denotes co-first authors (contributed equally to manuscript)

*Corresponding Author: Patrick Sullivan


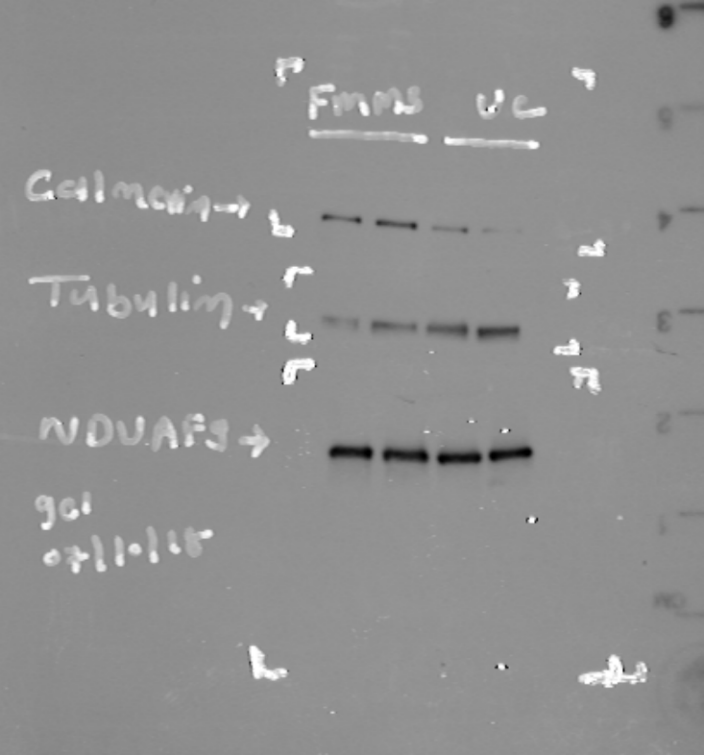

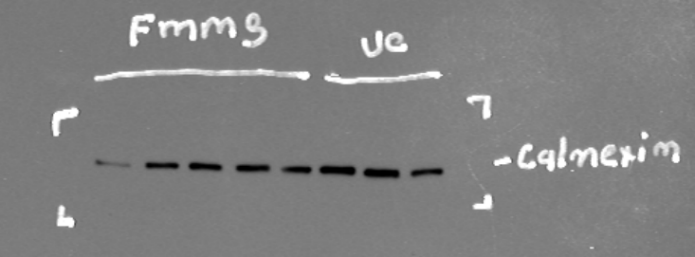

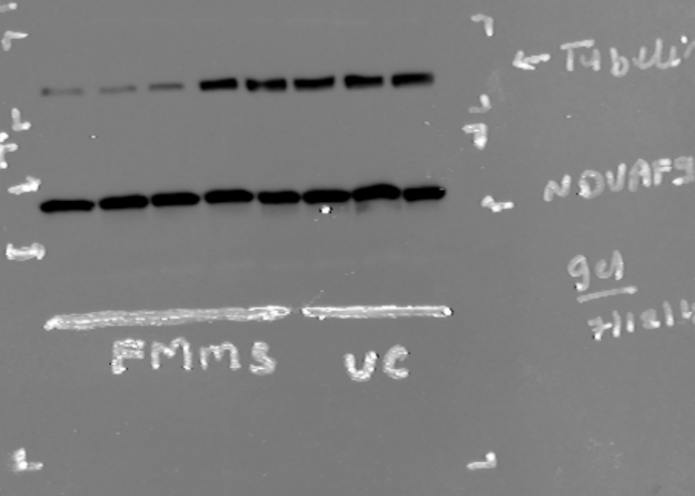


**Figure S1. (A)** Slot blot performed on markers of interest (calnexin, tubulin, and NDUAF9) with image exposure of 15 seconds. **(B)** Slot blots were performed in separate runs. These images were taken with an exposure time of 20 and 30 seconds, respectively. These markers are established in our laboratory with appropriate negative and positive controls. Red boxes are drawn around blots used in the full manuscript.


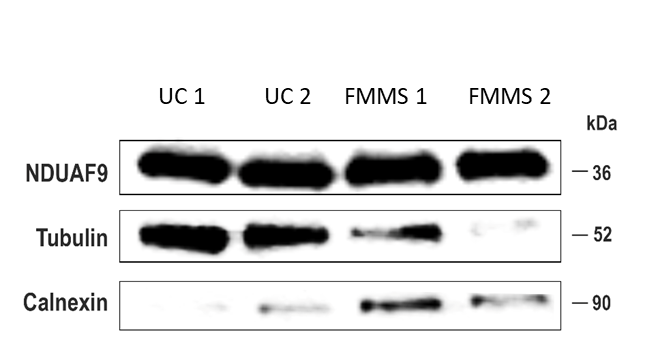


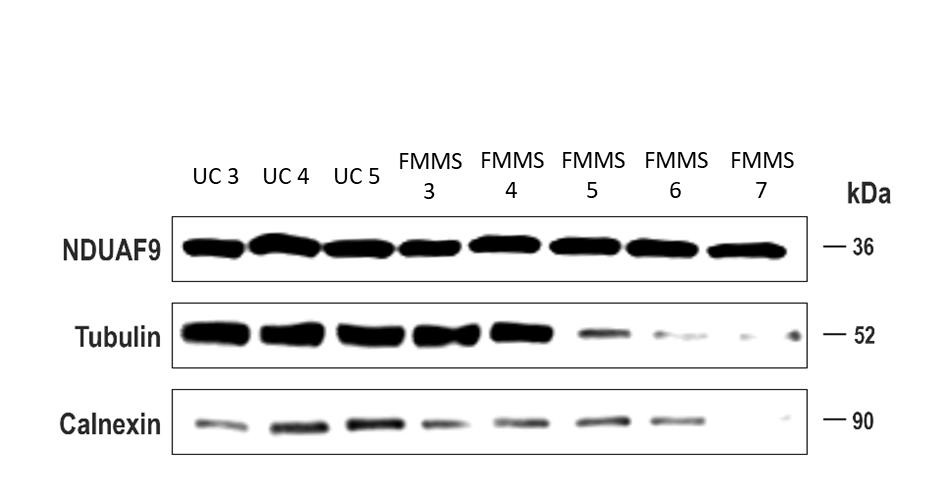


**Figure S2.** Processed full-length slot blots for markers of mitochondrial fraction purity after either UC or FMMS procedures. Slot blots were performed in separate runs. Red boxes are drawn around blots used in the full manuscript.
